# Real-Time High-Resolution OCT for Imaging Retinal and Choroidal Blood Flow

**DOI:** 10.1167/iovs.67.5.69

**Published:** 2026-05-27

**Authors:** Alessandro Invernizzi, Francesco Romano, Federico Corvi, Mariano Cozzi, SriniVas R. Sadda, K. Bailey Freund, Giovanni Staurenghi

**Affiliations:** 1 Eye Clinic, Department of Biomedical and Clinical Sciences, Ospedale Luigi Sacco, University of Milan, Milan, Italy; 2 Save Sight Institute, University of Sydney, Sydney, New South Wales, Australia; 3 Harvard Retinal Imaging Lab, Department of Ophthalmology, Massachusetts Eye and Ear, Harvard Medical School, Boston, Massachusetts, United States; 4 Department of Ophthalmology, University Hospital Zurich, University of Zurich, Zurich, Switzerland; 5 Department of Ophthalmology, David Geffen School of Medicine, University of California Los Angeles, Los Angeles, California, United States; 6 Vitreous Retina Macula Consultants of New York, New York, New York, United States; 7 Department of Ophthalmology, New York University Grossman School of Medicine, New York, New York, United States

**Keywords:** High-Res OCT, high-resolution, real-time, live OCT, flow assessment, particle motion

## Abstract

**Purpose:**

To evaluate the feasibility of visualizing intravascular moving particles within retinal and choroidal vessels using real-time high-resolution optical coherence tomography (High-Res OCT), a prototype device offering 3-µm axial resolution.

**Methods:**

In this cross-sectional study, 20 healthy eyes were imaged using both High-Res OCT and the standard SPECTRALIS HRA+OCT. A dedicated in-built research tool enabled ART-1 B-scan movie acquisition with real-time eye-tracking. Two masked graders qualitatively assessed visibility and direction of intravascular moving particles, and intergrader agreement was evaluated using Gwet's agreement coefficient 1 (AC1). Four additional patients with retinal vascular disorders (retinal artery occlusion, diabetic retinopathy, hypertensive retinopathy, and neovascular age-related macular degeneration) were imaged with High-Res OCT to explore illustrative clinical applications.

**Results:**

High-Res OCT demonstrated superior visualization of intravascular moving particles compared with standard OCT in both arteries (80% vs. 50%; *P* = 0.01) and veins (90% vs. 60%; *P* = 0.03). Intergrader agreement was high for High-Res OCT (AC1, 0.82–0.89) and moderate-to-substantial for standard OCT (AC1, 0.53–0.71). Flow direction assessment did not differ significantly between devices, although correct identification was numerically higher with High-Res OCT. In pathological eyes, real-time High-Res OCT enabled dynamic visualization of pulsatile and disturbed flow patterns and distinct intravascular motion across multiple vascular conditions.

**Conclusions:**

Real-time High-Res OCT enables direct, non-invasive visualization of intravascular moving particles within retinal and choroidal vessels and improves flow detectability compared with conventional OCT. This technique offers novel qualitative insights into ocular vascular dynamics and may guide future quantitative and translational applications.

Over the past decades, optical coherence tomography (OCT) has transformed clinical practice in ophthalmology and is now among the most widely used imaging modalities for assessing retinal and choroidal diseases.^1^^–^^3^ More recently, OCT angiography (OCTA) has expanded these capabilities by enabling non-invasive visualization of retinal and choroidal vasculature through motion contrast from sequential, densely acquired OCT scans.^4^^,^^5^ Although OCTA has important applications, the direct visualization and quantification of blood flow and other dynamic structures within retinal tissue remain significant challenges.

To address this limitation, several research groups have explored alternative solutions, including Doppler OCT,^6^ variable interscan scan time analysis (VISTA) OCTA,^7^ and fringe washout phenomena in real-time OCT,^8^ to infer temporal blood flow dynamics. Real-time OCT assessment, characterized by the continuous acquisition of consecutive B-scans, has recently gained momentum,^9^ especially in surgical settings, where it enables visualization of dynamic tissue structures and procedures.^10^ However, although OCT images are usually improved by averaging several sequential B-scans, in real-time OCT this comes at the cost of reduced frame rate, an undesirable trade-off for dynamic imaging. As such, overall image quality depends largely on the signal-to-noise ratio (SNR) of individual B-scans and the lateral and axial resolution of the device.^11^ SNR is influenced by the clarity of ocular media and is notably reduced by scattering and absorption along the optical path, such as lens opacities.^12^ Lateral resolution in clinical OCT systems is constrained by the small beam diameter at the pupil and ocular aberrations.^3^ Similarly, conventional axial resolution (∼7 µm) remains insufficient for subcellular visualization. These limitations have hindered the broader adoption of real-time OCT in clinical settings.^9^

In March 2021, Heidelberg Engineering (Heidelberg, Germany) introduced a high-resolution spectral-domain OCT system (High-Res OCT). This investigational device achieves an axial resolution of ∼3 µm in tissue by shifting the central wavelength, expanding the spectral bandwidth,^13^ and increasing the incident optical power at the pupil to compensate for the associated SNR loss.^12^ High-res OCT has shown improved test–retest reliability in retinal layer segmentation and enhanced qualitative visualization of cellular and subcellular retinal structures.^14^^–^^18^

Due to its unique characteristics, High-Res OCT may enhance the assessment of vascular flow in both static and real-time settings, thereby offering new insights into retinal and choroidal vascular physiology. The aim of this study was to investigate the visualization of intravascular moving particles—likely representing groups of blood cells or their scattering signatures—within retinal vessels using the High-Res OCT in real time and to compare the resulting video sequences with those acquired using the standard Heidelberg SPECTRALIS HRA+OCT system.

## Methods

### Study Design and Participants

This cross-sectional study was conducted at the Eye Clinic of Ospedale Luigi Sacco (University of Milan, Milan, Italy) between January and December 2024. The research adhered to the tenets of the Declaration of Helsinki, and written informed consent was obtained from all participants prior to inclusion. Ethical approval was granted by the University of Milan Ethics Committee (protocol no. 19.22).

A total of 20 healthy volunteers were recruited from the medical staff of Ospedale Luigi Sacco. Exclusion criteria were (1) poor fixation, (2) significant media opacities, (3) refractive error exceeding ±6 diopters, and (4) any history of ocular inflammation or ophthalmic surgery. One eye per participant was included in the analysis. If both eyes met eligibility criteria, the right eye was selected. In addition, four patients were included who had retinal disorders, including retinal artery occlusion (RAO), diabetic retinopathy (DR), hypertensive retinopathy, and neovascular age-related macular degeneration (nAMD). These patients were not part of the primary comparative analysis (flow visualization with High-Res OCT vs. SPECTRALIS HRA+OCT) but were imaged exclusively with the High-Res OCT device to explore its potential for visualizing blood flow in the setting of vascular pathology.

### High-Res OCT Versus SPECTRALIS HRA+OCT

The High-Res OCT is a novel investigational device developed by Heidelberg Engineering that offers an enhanced axial resolution of 3 µm in tissue, compared to the 7-µm resolution of the standard SPECTRALIS HRA+OCT system. This improvement is achieved by shifting the central wavelength from 880 to 853 nm and expanding the spectral bandwidth from 50 to 137 nm, while maintaining a comparable lateral resolution of ∼14 µm.^19^^,^^20^ To compensate for the larger bandwidth—and the associated reduction in SNR due to higher axial resolution^12^—the incident optical power was increased from 1.2 to 2.2 mW. The system employs a superluminescent diode-based light source, with ocular exposure evaluated by the manufacturer in accordance with established international safety standards (ANSI Z80.36 and IEC 60825-1) and remaining well within applicable limits for clinical OCT imaging.

### Acquisition Protocol

#### Flow Assessment in Healthy Eyes

**Figure 1.**
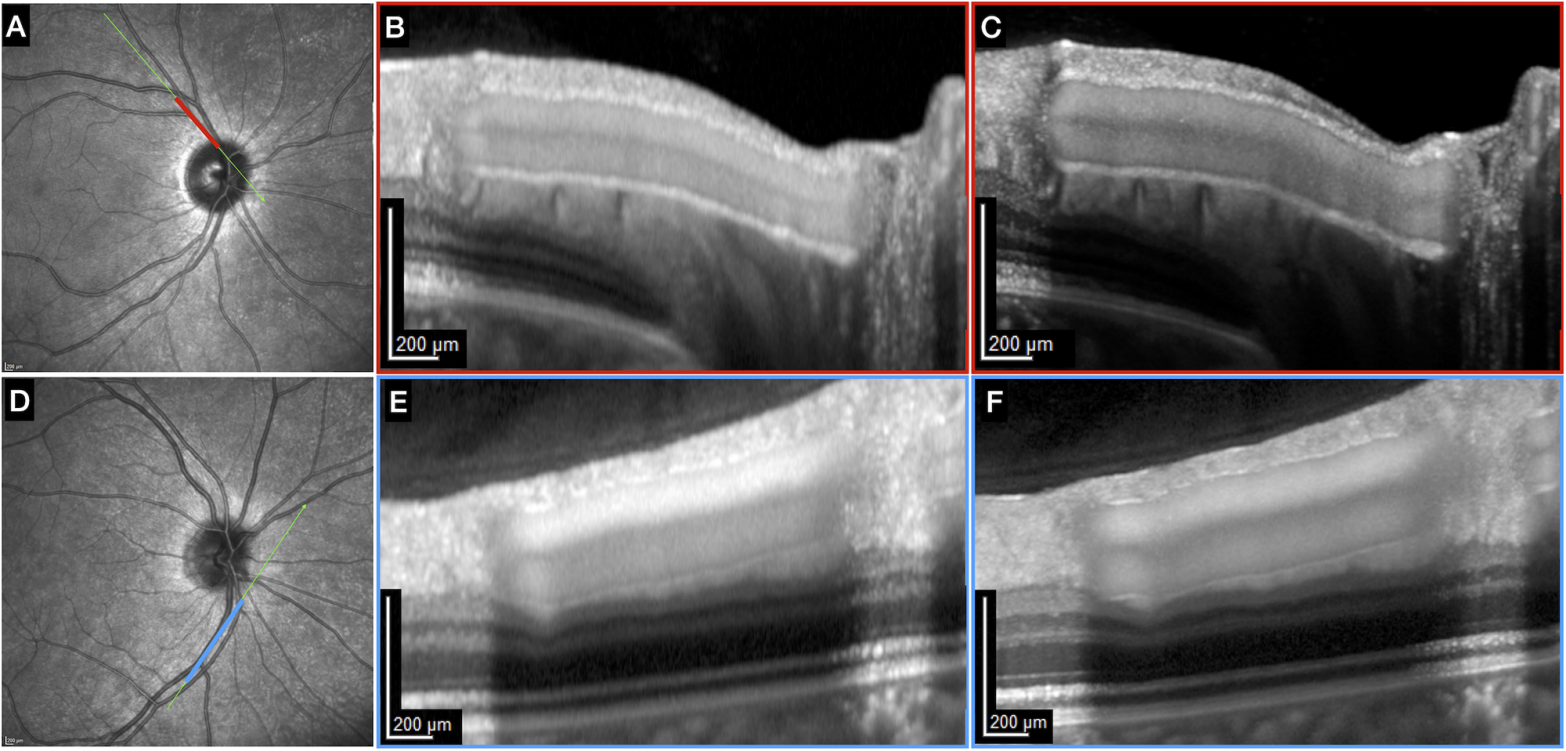
Representative imaging protocol in healthy subjects using the standard SPECTRALIS OCT and the investigational High-Res OCT. A single 10° OCT B-scan was positioned co-axially along a main branch of the central retinal artery (**A**–**C**) and the central retinal vein (**D**–**F**), centered on the selected vessel. Using the near-infrared reflectance image (**A**, **D**) as a reference, scans were performed with the standard device (**B**, **E**) and then repeated in the exact same location with the High-Res OCT device (**C**, **F**) using the scan/rescan function of the HEYEX software (version 1.10.4.0) The increased details of the images obtained with the High Res device is particularly evident in these examples.

Following pupil dilation with 0.5% tropicamide plus 2.5% phenylephrine, each healthy subject underwent real-time single B-scan acquisitions using both the standard SPECTRALIS HRA+OCT and the investigational High-Res OCT. For each eye, a 10° high-resolution single-line scan was positioned co-axially with one of the main branches of the central retinal artery and central retinal vein, centered over the selected vessel (Fig. 1). Vessel selection was informed by preliminary live OCT observations in which intravascular motion was visually detected during real-time High-Res OCT acquisition. For each arterial and venous branch, up to three acquisitions were obtained to ensure adequate technical quality. For analysis, the best-quality ART-1 movie was selected; in 15 of 20 arteries and 17 of 20 veins, the first acquired movie was retained, indicating that repeated acquisitions were not systematically required. During this exploratory phase, live OCT images were documented by screen recording the live OCT window of the Heidelberg Eye Explorer software (HEYEX 1.10.4.0) using an external application (Icecream Screen Recorder 7.43) while patients maintained steady fixation. The “live” OCT display consisted of continuous B-scans (512 A-scans per B-scan) acquired at an A-scan rate of 85 kHz, corresponding to a theoretical maximum B-scan acquisition rate of approximately 166 Hz. A subset of these B-scans was rendered in real time by the HEYEX software for on-screen visualization; however, the effective display rate is constrained by software and hardware limitations and does not necessarily reflect the native acquisition speed. Following this exploratory phase, a single averaged B-scan was acquired using the Automatic Real Time (ART) function, exported as an .E2E file, and imported into the High-Res OCT system to guide re-acquisition at the same anatomical location.

To overcome the inherent limitations of screen-based recording—including reliance on external screen-capture software, screen-dependent resolution, and lack of eye-tracking—a dedicated in-built research tool was developed in collaboration with Heidelberg Engineering. This tool, currently investigational and not commercially available, enables ART-based control of the effective frame rate by determining the number of B-scans averaged per stored frame. For this study, ART was set to 1, resulting in an effective acquisition rate without any frame averaging. The tool operates in eye-tracking mode, which helps maintain spatial alignment during acquisition but may introduce temporal gaps when individual B-scans are discarded due to tracking delays or misalignment. These interruptions are precisely recorded using embedded time stamps for each frame within the movie file. Because the analysis was qualitative and did not aim to quantify flow velocity or continuity, temporal gaps did not affect grading. Movie sequences were reviewed in their entirety, and visibility of moving particles and flow direction were assessed independently of frame-to-frame continuity. To characterize temporal fidelity, we retrospectively analyzed per-frame DICOM time stamps from all arterial and venous ART-1 recordings. The nominal inter-frame interval was 11 ms (∼90.9 fps), whereas the effective mean frame rate was 74.7 fps for arterial recordings and 78.2 fps for venous recordings. True temporal discontinuities, defined as inter-frame gaps ≥ 55 ms, consistent with eye-tracking correction events, occurred in fewer than 5% of inter-frame transitions in both groups (Supplementary Fig. S1, Supplementary Table S1). In case of pulsatile or intermittent motion, flow direction was determined based on the predominant direction of particle displacement during the sequence; cases in which direction could not be confidently inferred were graded as unclear. This research tool was implemented into the HEYEX software for both OCT systems, allowing ART-1 B-scan movies to be exported in .E2E format and re-acquired at the same location using the High-Res OCT.

All ART-1 B-scan movies acquired with the two OCT systems were independently reviewed by two medical retina fellowship-trained ophthalmologists (FR, FC), with discrepancies adjudicated by a senior grader (AI). Given the exploratory study design, primary endpoints were qualitative feasibility measures selected to establish proof-of-concept prior to developing objective quantitative metrics. Each acquisition was qualitatively evaluated using the HEYEX review software for the following parameters:

• Visibility of moving particles within the vessel lumen was graded as visible, not visible, or unclear.
• Flow direction within the vessel was assessed by evaluating the direction of particle motion on real-time OCT, with the graders masked to the anatomical orientation of the vessel; correctness was determined by concordance with the expected physiological flow direction based on vessel identity and co-registered near-infrared images.

#### Flow Assessment in Diseased Eyes

**Figure 2.**
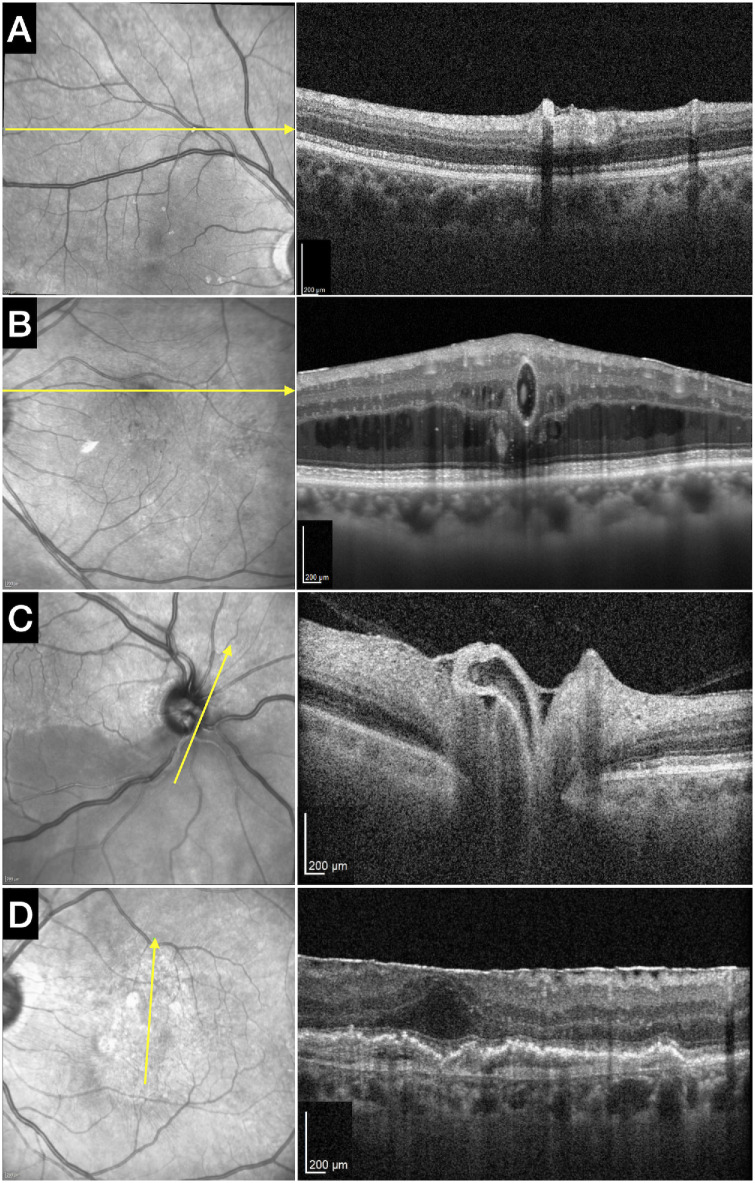
Representative single frames from real-time High-Res OCT acquisitions across pathological retinal and choroidal vessels. Scan orientation and length were customized according to lesion morphology to optimize visualization of intravascular motion and flow dynamics. (**A**) Hypertensive retinopathy with calcific embolus. (**B**) Retinal microaneurysms (diabetic retinopathy). (**C**) Retinal artery occlusion. (**D**) Type 1 macular neovascularization (nAMD).

In patients with retinal disease, blood flow dynamics within pathological retinal and choroidal vessels were assessed using the same pupil-dilated imaging protocol, with the primary aim of demonstrating the potential clinical utility of “live” High-Res OCT imaging. For this purpose, imaging was performed exclusively with the High-Res OCT using the specifically developed research tool. The B-scan acquisition pattern (e.g., position, orientation, length) was manually tailored by the operator based on the location and morphology of the lesion of interest. For example, horizontal B-scans were selected for retinal aneurysms, and scans were oriented longitudinally to follow the axis of stenosed or dilated vessels, as well as the largest dimension of macular neovascularization (MNV). Representative examples of acquisition strategies in diseased eyes are shown in Figure 2.

### Statistical Analysis

Statistical analyses were conducted using RStudio 2023.12.0+369 (RStudio, PBC; Boston, MA, USA). Data are reported as mean ± standard deviation, median (interquartile range), and frequency (as a percent), as appropriate. No formal a priori sample size calculation was performed consistent with the exploratory nature of the study and the absence of prior data from which to derive reliable estimate of the expected effect size.

Interrater agreement for each parameter was assessed separately for the two devices using Gwet's AC1 coefficient (95% confidence intervals [CIs]), given the small sample size. Agreement was reported numerically, with higher AC1 values indicating stronger interrater agreement. Comparisons between the sensitivities of the two devices in detecting each parameter were performed using McNemar's test and reported as χ^2^ values. All tests were two sided, with significance defined as *P* < 0.05.

## Results

A total of 40 retinal vessels (20 arteries and 20 veins) from 20 eyes of 20 healthy subjects (11 females, 55%) were imaged using both the SPECTRALIS HRA+OCT and High-Res OCT and included in the primary analysis. The mean age of participants was 34.4 ± 5.6 years. All subjects were White non-Hispanic (20 of 20, 100%).

### Analysis of Blood Flow in Healthy Eyes

When assessing the visibility of moving particles within the vessel lumen, interrater agreement was higher for High-Res OCT (Gwet's AC1, 0.82–0.89) than for the standard SPECTRALIS HRA+OCT (Gwet's AC1, 0.53–0.71). Moving particles were more frequently graded as visible using High-Res OCT compared to standard OCT, both in arteries (16/20 vs. 10/20 acquisitions, 80% vs. 50%; χ^2^ = 6.0; *P* = 0.01) and in veins (18/20 vs. 12/20 acquisitions, 90% vs. 60%; χ^2^ = 4.5, *P* = 0.03). Supplementary Video S1 shows a representative example.

For flow direction assessment, interrater agreement was higher with High-Res OCT (Gwet's AC1, 0.42–0.53) than with SPECTRALIS HRA+OCT (Gwet's AC1, 0.23–0.27). Flow direction was more often correctly identified using High-Res OCT than SPECTRALIS HRA+OCT in both arteries (60% vs. 40%; absolute difference, +20% points; 95% CI, −6.3 to 46.3) and veins (65% vs. 45%; absolute difference, +20% points; 95% CI, −6.3 to 46.3); however, these differences did not reach statistical significance (χ^2^ = 2.0 and χ^2^ = 2.0, respectively; both *P* > 0.05).

### Analysis of Eyes With Retinal Disorders

A summary of the illustrative cases with retinal disorders is provided in the Table.

**Table.** Illustrative Applications of Real-Time High-Res OCT in Retinal Diseases

Diagnosis	Key Findings on High-Res OCT	Interpretative Value
Retinal artery occlusion	Narrowed arterial lumen partially obstructed by an intraluminal hyperreflective structure with sparse intraluminal moving particles Adjacent venous flow showing intermittent, pulsatile “stop-and-go” motion	Suggests severe arterial flow restriction with secondary alterations in venous hemodynamics. Enables direct visualization of residual intravascular motion and pulsatile venous flow in acute ischemia
Retinal microaneurysms (diabetic retinopathy)	Active particle motion within hyperreflective regions of the aneurysm lumen and intermittent motion within hyporeflective areas not showing OCTA signal	Supports the presence of markedly slow intraluminal flow below the OCTA detection threshold Complements OCTA by revealing dynamic flow heterogeneity within microaneurysms
Hypertensive retinopathy with calcific embolus	Irregular, non-laminar venous flow at arteriovenous crossings	Highlights disturbed venous hemodynamics at compression sites
	Preserved arterial flow at the site of a previously documented calcific embolus	Suggests possible embolus migration or extrusion with vessel recanalization, consistent with prior reports
Type 1 macular neovascularization (nAMD)	Clear differentiation between flow-positive vascularized and flow-negative fibrotic components Visualization of neovessels crossing Bruch's membrane with identifiable inflow and outflow directions	Provides additional insight into 3D MNV architecture and neovascular flow organization beyond conventional structural OCT and OCTA

#### Case 1. Retinal Artery Occlusion

**Figure 3.**
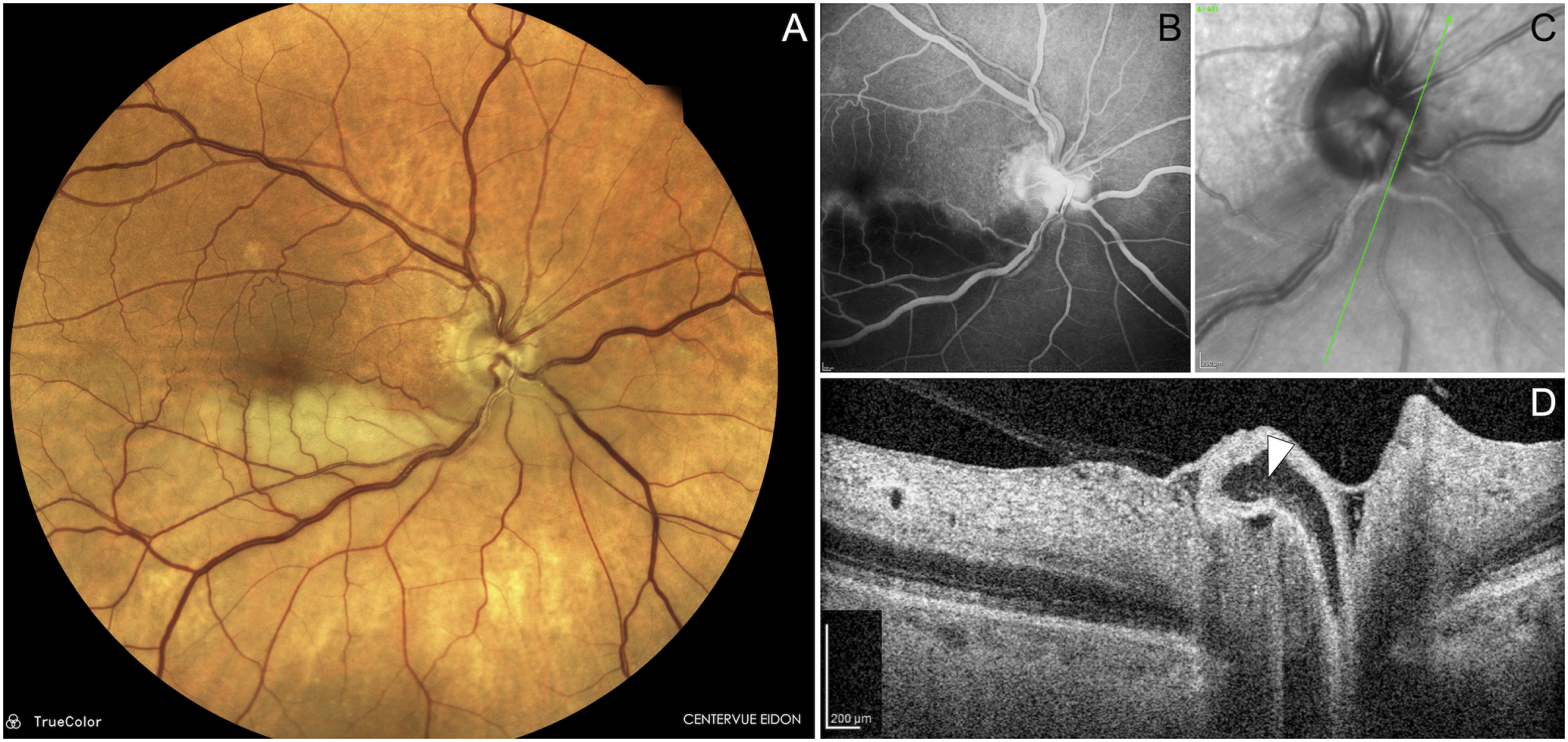
Acute retinal artery occlusion. (**A**, **B**) Fluorescein angiography demonstrates markedly reduced arterial flow and delayed perfusion in the inferior retina. (**C**, **D**) A single frame from a real-time High-Res OCT scan acquired along the affected arterial branch shows a predominantly hyporeflective arterial lumen, with scattered hyperreflective intravascular moving particles (*white arrowhead*), likely representing residual intraluminal motion within a partially obstructed vessel.

This case features a patient with acute RAO diagnosed 36 hours before imaging. Fluorescein angiography revealed markedly reduced arterial flow and delayed perfusion in the inferior retina (Figs. 3A, 3B). High-Res OCT single-line scans of the occluded retinal artery revealed: (1) a narrowed arterial lumen partially obstructed by an intraluminal hyperreflective structure, and (2) few floating intraluminal particles within the patent segment, likely representing blood cell aggregates or their scattering signatures. These particles exhibited rapid and turbulent motion on live imaging, consistent with flow restriction through the narrowed vessel (Figs. 3C, 3D). Live High-Res OCT imaging of the adjacent vein provided a clearer visualization of intraluminal moving elements progressing centripetally in a “stop-and-go” pattern (Supplementary Video S2). This dynamic likely reflects pulsatile venous flow due to impaired arterial perfusion.^21^ Intravascular moving particles appeared to stagnate within the venous lumen during the diastolic phase and slowly resumed motion toward the optic nerve head during systole.

#### Case 2. Retinal Microaneurysms

**Figure 4.**
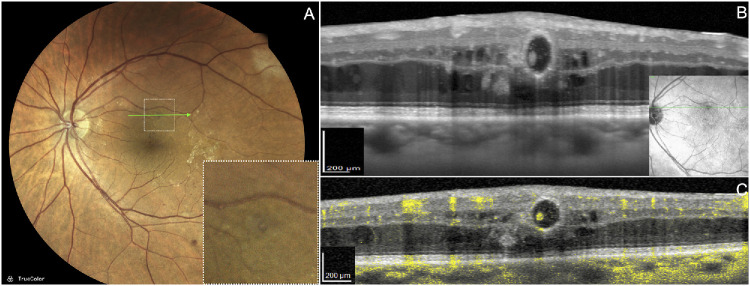
(**A**) Non-proliferative diabetic retinopathy with a large microaneurysm located superior to the fovea. (**B**) Structural High-Res OCT revealed a predominantly hyporeflective aneurysmal lumen with a focal hyperreflective component. (**C**) Corresponding OCT angiography B-scan shows flow signal confined to the hyperreflective region, suggesting markedly slow or heterogeneous intraluminal flow.

In this patient with non-proliferative DR, multiple microaneurysms were observed at the posterior pole, including one prominently located superior to the fovea. Structural OCT revealed heterogeneous intraluminal reflectivity, and OCTA showed flow signal only within the hyperreflective regions (Fig. 4). Live High-Res OCT imaging confirmed active particle motion within the hyperreflective portion and revealed intermittent motion within the hyporeflective areas of the lumen (Supplementary Video S3). These findings are consistent with markedly slow intraluminal flow, which may fall outside the temporal sensitivity window of OCTA, resulting in absent or reduced decorrelation signal despite ongoing perfusion. This interpretation supports computational fluid dynamics models predicting regions of reduced flow velocity and particle displacement within microaneurysms.^22^

#### Case 3. Hypertensive Retinopathy and Calcific Embolus

**Figure 5.**
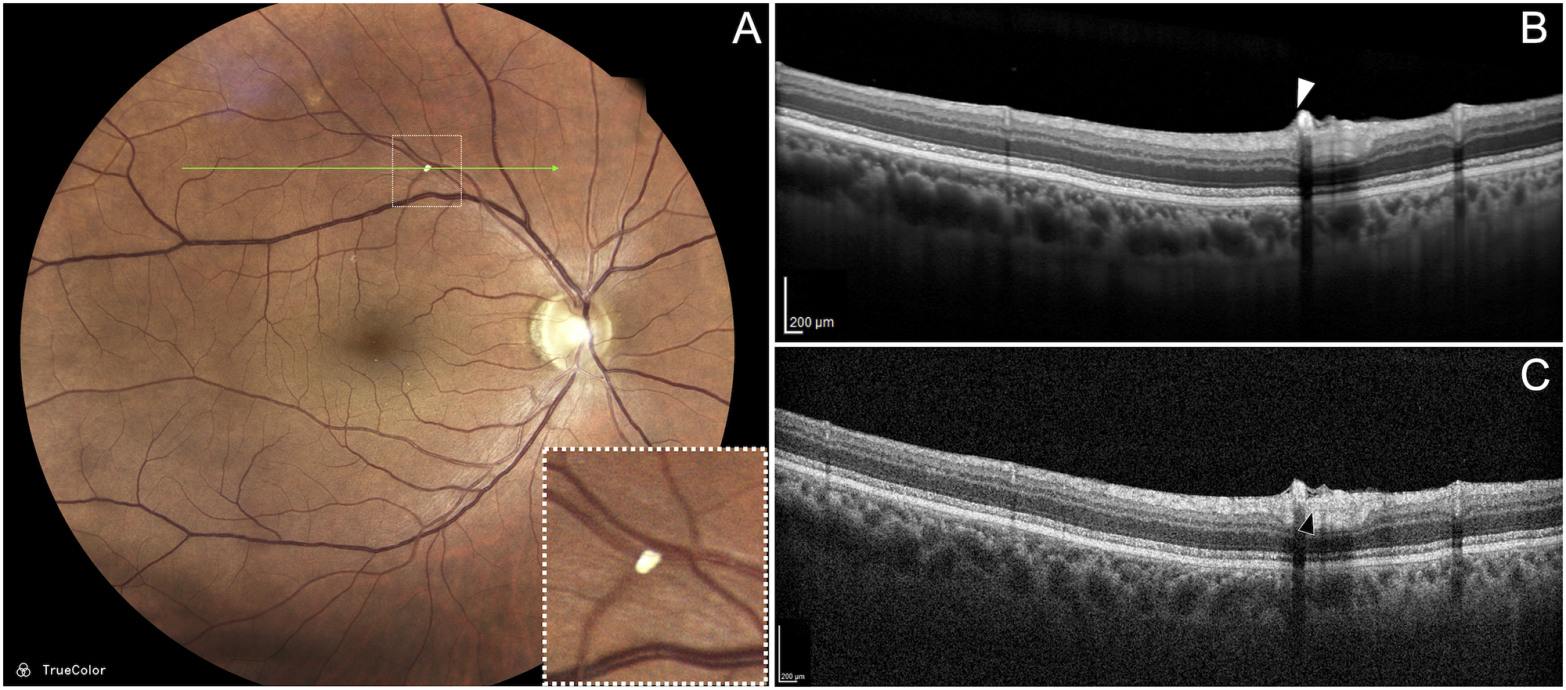
(**A**) Mild hypertensive retinopathy with compressive arteriovenous crossings and a calcific embolus at an arterial bifurcation along the superotemporal arcade. (**B**) High-Res OCT B-scan reveals the embolic material situated on a different plane from the arterial lumen, protruding beyond the vessel wall (*white arrowhead*). (**C**) A single frame from the real-time High-Res OCT shows intravascular moving signal beneath the embolus (*black arrowhead*), confirming preserved downstream flow.

This case features a patient with mild hypertensive retinopathy with compressive arteriovenous crossings and a calcific embolus at an arterial bifurcation along the superotemporal arcade (Fig. 5). Real-time High-Res OCT demonstrated irregular, non-laminar venous flow at the arteriovenous crossing, in contrast to the smooth, continuous flow pattern observed in normal veins imaged with the same technique and thus supporting a hemodynamic mechanism for endothelial damage and potential branch retinal vein occlusion (Supplementary Video S4).^23^ In contrast, arterial flow appeared preserved at the site of previously documented calcific embolus (Supplementary Video S5). Although the exact intraluminal versus extraluminal location of the embolic material cannot be definitively established with the available imaging, this finding is consistent with prior reports describing embolus migration or extrusion with subsequent vessel recanalization.^24^

#### Case 4. Macular Neovascularization in nAMD

**Figure 6.**
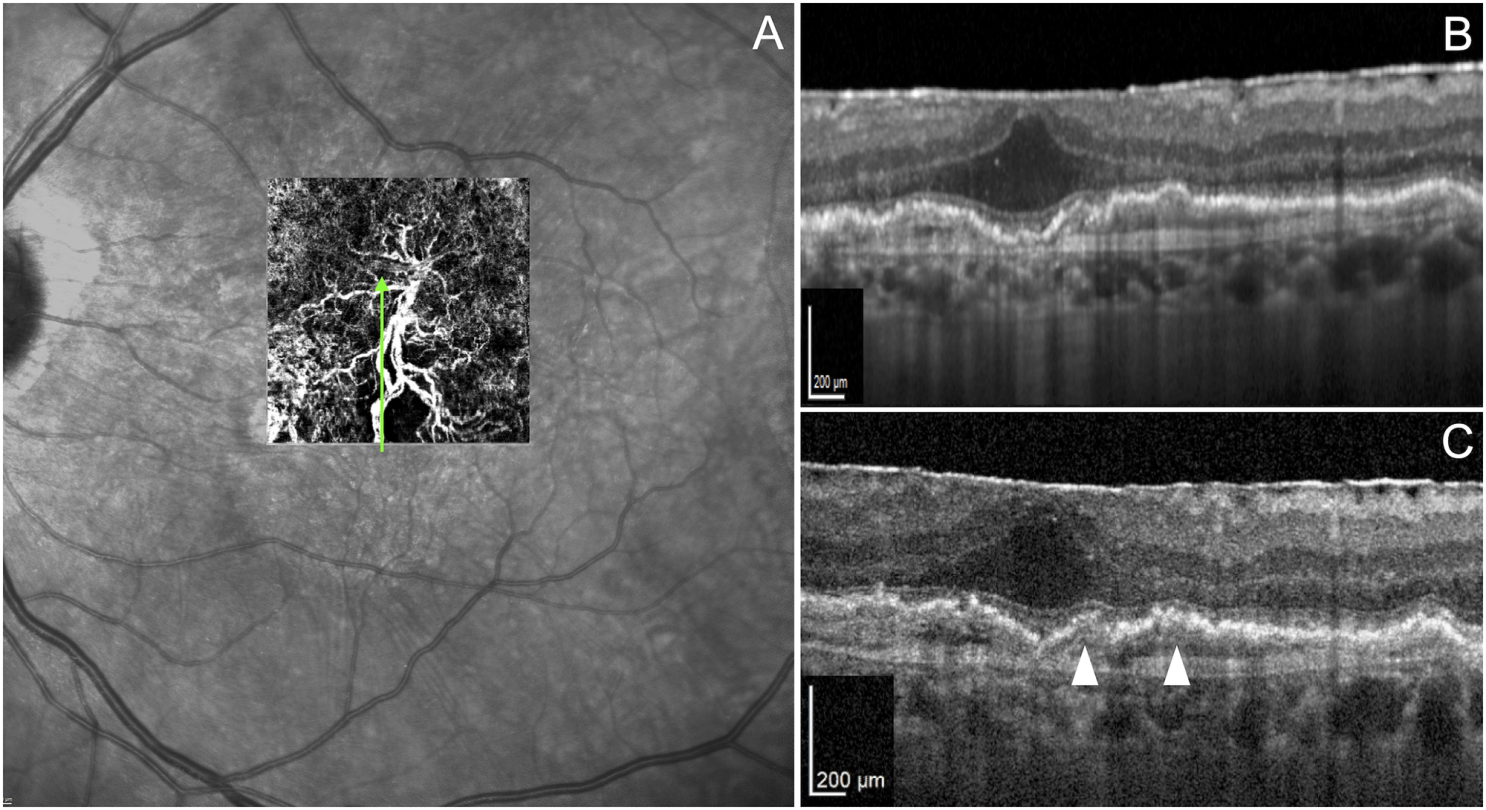
Imaging features of type 1 MNV. (**A**) En face OCT angiography reveals an extensive subretinal pigment epithelium (RPE) neovascular complex. (**B**) Structural B-scan OCT angiography shows two distinct hyperreflective structures between the RPE and Bruch membrane, consistent with fibrotic and vascular elements of the MNV. (**C**) A single frame from real-time High-Res OCT shows intravascular moving particles within the vascularized component, aiding differentiation between flow-positive neovascular tissue and adjacent fibrotic regions.

This last case shows a patient with type 1 MNV, diagnosed via OCTA. Although standard imaging provided sufficient diagnostic information (Figs. 6A, 6B), live High-Res OCT offered additional insights into the 3D structure of the neovascular membrane and its relationship with the underlying choroid. Live visualization of moving particles enabled differentiation between the vascularized (flow-positive, top) and fibrotic (flow-negative, bottom) components of the MNV (Fig. 6C; Supplementary Video S6). Additionally, small intravascular particles were detected in vessels crossing Bruch's membrane. Flow direction analysis distinguished choroid-derived neovascular arteries supplying the lesion from draining venules. This case underscores the added value of real-time High-Res OCT in characterizing MNV architecture and flow dynamics.

## Discussion

In this study, we introduced a novel approach for visualizing intravascular particle motion within retinal and choroidal vessels using real-time High-Res OCT imaging. This first-in-human application was made possible by combining real-time OCT, recently implemented in macular surgery, with the enhanced axial resolution offered by the investigational Heidelberg High-Res OCT device.

To date, OCTA and Doppler OCT represent the most widely used non-invasive techniques for assessing retinal and choroidal blood flow. OCTA enables high-resolution visualization of the microvascular architecture but relies on indirect, static flow detection based on decorrelation between sequential scans, providing no information on flow speed or direction and making it difficult to distinguish hypoperfusion from complete ischemia.^4^ Conversely, Doppler OCT offers quantitative flow velocity measurements but is limited by angle dependence, signal noise, and complex interpretation, particularly in the presence of physiological pulsations.^6^^,^^21^

The approach introduced in this study addresses several of the limitations associated with existing imaging modalities and offers a novel methodology for the assessment of retinal and choroidal blood flow. Albeit not yet widely accessible, real-time High-Res OCT provides distinct advantages as a dynamic imaging technique. It enables direct visualization of moving particles within the vessel lumen and offers qualitative information regarding the presence, relative quantity, and, to some extent, the direction of flow. These intravascular moving signals most likely reflect the motion of blood cell aggregates and dynamic scattering interfaces within the flowing blood column, which become detectable due to the improved axial resolution and contrast of the High-Res OCT system. Our comparative analysis demonstrated that High-Res OCT, acquired at the same A-scan rate (85 kHz, corresponding to a B-scan rate of approximately 166 Hz), showed numerically higher directional accuracy than standard SPECTRALIS HRA+OCT in most subjects. Because acquisition speed was matched between devices, this improvement is most plausibly attributable to the superior axial resolution of the High-Res OCT, which enhances contrast between intravascular moving particles and surrounding tissue. Nevertheless, inter-grader agreement for flow direction was only moderate with High-Res OCT (AC1, 0.42–0.53) and poor-to-fair with standard OCT (AC1, 0.23–0.27), and directional performance should therefore be interpreted cautiously. Temporal sampling remains a key limitation of real-time OCT.^25^ When intravascular moving particles travel faster than the effective frame rate, tracking the trajectory of individual moving elements becomes challenging, and they may appear at spatially disjointed positions across consecutive frames, producing a “jumping” appearance that complicates directional inference. Pulsatile or intermittent motion may further reduce grader confidence. Although higher axial resolution and faster B-scan rates likely act synergistically, the present study was not designed to disentangle their individual contributions. Future systems combining ultrahigh axial resolution with faster B-scan acquisition may further improve flow direction assessment.

Although OCTA remains advantageous for generating widefield, depth-resolved vascular maps navigable through coronal reconstructions,^26^ the real-time OCT approach used here is restricted to localized assessment within a single B-scan plane. Although flow information can be visualized across retinal and choroidal depths within the same scan, each acquisition must be manually aligned with the vessel of interest, precluding comprehensive flow or volumetric mapping. The in-built research tool developed by Heidelberg Engineering—designed to generate same-location, consecutive B-scan movies with active eye-tracking—was instrumental in acquiring real-time OCT recordings. Without continuous eye tracking, maintaining a stable B-scan aligned to a specific vascular structure for the duration required to capture a movie would be virtually impossible. It is important to note that the software temporarily halts B-scan acquisition if the eye deviates beyond a defined angular threshold from its initial position and automatically resumes once alignment is re-established. Although this feature significantly enhances image stability during acquisition, it also introduces temporal discontinuities, resulting in final recordings composed of multiple short segments stitched together. Despite these limitations, the ability to visualize intravascular flow dynamics in real time offers substantial advantages for investigating retinal and choroidal diseases, both in research and clinical practice.

The preliminary cases included in this study illustrate this potential. For example, in a scan of a retinal arterial microaneurysm, live High-Res OCT revealed the different contents of the bulging lumen and their relative movements, with intravascular particle motion restricted to a specific portion of the lesion and the remaining volume occupied by plasma only occasionally accompanied by cellular elements. In another case, analysis of a type 1 MNV allowed clear differentiation between the flow-positive vascular component of the lesion and the surrounding fibrotic tissue. Live High-Res OCT also enabled direct visualization of neovascular vessels originating from the choroid, crossing Bruch's membrane, and contributing to the pathological network. These observations may enhance our understanding of “neo-choriocapillaris” involvement in type 1 MNV and open new avenues for investigating flow dynamics in other neovascular subtypes, such as retinal angiomatous proliferation (type 3) and polypoidal choroidal vasculopathy.^27^^,^^28^ The analysis of retinal vessels in the context of vascular occlusions was even more striking. Real-time OCT imaging in a case of retinal artery occlusion revealed two key findings: (1) the complete absence of moving elements within the affected arterial branches, and (2) delayed, pulsatile flow within adjacent veins. The ability to detect residual venous flow in such scenarios is clinically significant and typically requires invasive imaging modalities such as fluorescein angiography.^29^ This is because the flow may be too slow to generate detectable signal on OCTA, yet it provides valuable information about the actual perfusion status of the retina.^4^ Similarly, the assessment of arteriovenous crossings and embolic lesions in a patient with systemic hypertension and hypercholesterolemia highlighted the potential of real-time OCT to enhance the visualization of early hemodynamic disturbances preceding vascular occlusion.^30^ This improved capacity to detect subtle alterations in flow dynamics may offer critical insights into the pathogenesis of these conditions, potentially facilitating earlier diagnosis and deeper understanding of the mechanisms underlying vascular occlusive events.

This novel imaging approach is exploratory and, as such, carries some important limitations. First, the sample size was limited, which constrained the statistical analysis to basic measures such as intergrader agreement and device comparisons between standard OCT and High-Res OCT. Second, the primary endpoints were qualitative and operator dependent, lacking objective, quantitative validation or standardized flow metrics for detection, directionality, and velocity assessment, thus limiting its current reproducibility and interpretability. Third, vessel selection in healthy eyes was informed by live imaging and based on acquisition quality rather than a prespecified branch-selection scheme, potentially introducing selection bias and overestimating flow detectability. Fourth, only a small number of pathological conditions were included, with the intent to illustrate potential applications rather than to provide generalizable clinical data. Fifth, temporal fidelity of the recorded movies was inherently influenced by eye-tracking behavior. Although the effective mean frame rate remained high (74.7–78.2 fps against a nominal cadence of ∼90.9 fps), true temporal discontinuities (inter-frame gaps ≥ 55 ms) occurred in fewer than 5% of transitions and may still have affected qualitative assessment in some recordings. Sixth, the narrowly selected study population limits generalizability to more diverse cohorts and clinically relevant imaging conditions, including mild media opacities and broader refractive ranges. Finally, our real-time High-Res OCT approach is currently applicable only to large and medium-sized vascular structures and does not yet permit reliable assessment of smaller vascular plexuses.

Despite these limitations, the present findings provide a conceptual framework for future quantitative developments. Higher B-scan acquisition rates may enable frame-to-frame displacement analysis of intravascular moving particles, allowing estimation of relative flow velocity and directionality. Integration of real-time High-Res OCT with established flow-sensitive techniques, such as OCTA or Doppler OCT, and prospective matched-region multimodal comparisons will be important to define the incremental value of this approach. In this context, the combination of real-time acquisition with the improved axial resolution of High-Res OCT (∼3 µm in tissue) may provide complementary cross-sectional information on local intravascular motion that is not directly accessible with conventional OCT systems. Continued advances in scanning speed, motion correction, and automated tracking will be essential to translate this technique from qualitative assessment toward reliable quantitative analysis.

To conclude, by integrating the enhanced resolution of the new Heidelberg High-Res OCT with real-time imaging enabled by a dedicated research tool, we present a novel method for visualizing intravascular moving particles within retinal and choroidal vessels, without the need for dye injection. Although the technique is subject to inherent technical limitations, it offers invaluable advantages for assessment of the ocular circulation. Further studies are warranted to refine this approach, develop objective flow quantification tools, and explore its broader applications in both clinical and research settings.

## Supplementary Material

Supplement 1

Supplement 2

Supplement 3

Supplement 4

Supplement 5

Supplement 6

Supplement 7

Supplement 8
