# Supplementary material for: Real-Time High-Resolution OCT for Imaging Retinal and Choroidal Blood Flow
Source: Invest Ophthalmol Vis Sci. 2026 May 27;67(5):69. doi: 10.1167/iovs.67.5.69 (PMC13221891; doi:10.1167/iovs.67.5.69)
Supplement: Supplement 1 [file iovs-67-5-69_s001.docx]

**Supplementary Figure 1. Inter-frame timing analysis for one representative arterial (A) and one representative venous (B) ART-1 B-scan recording acquired with the Heidelberg Spectralis system (401 frames each).**


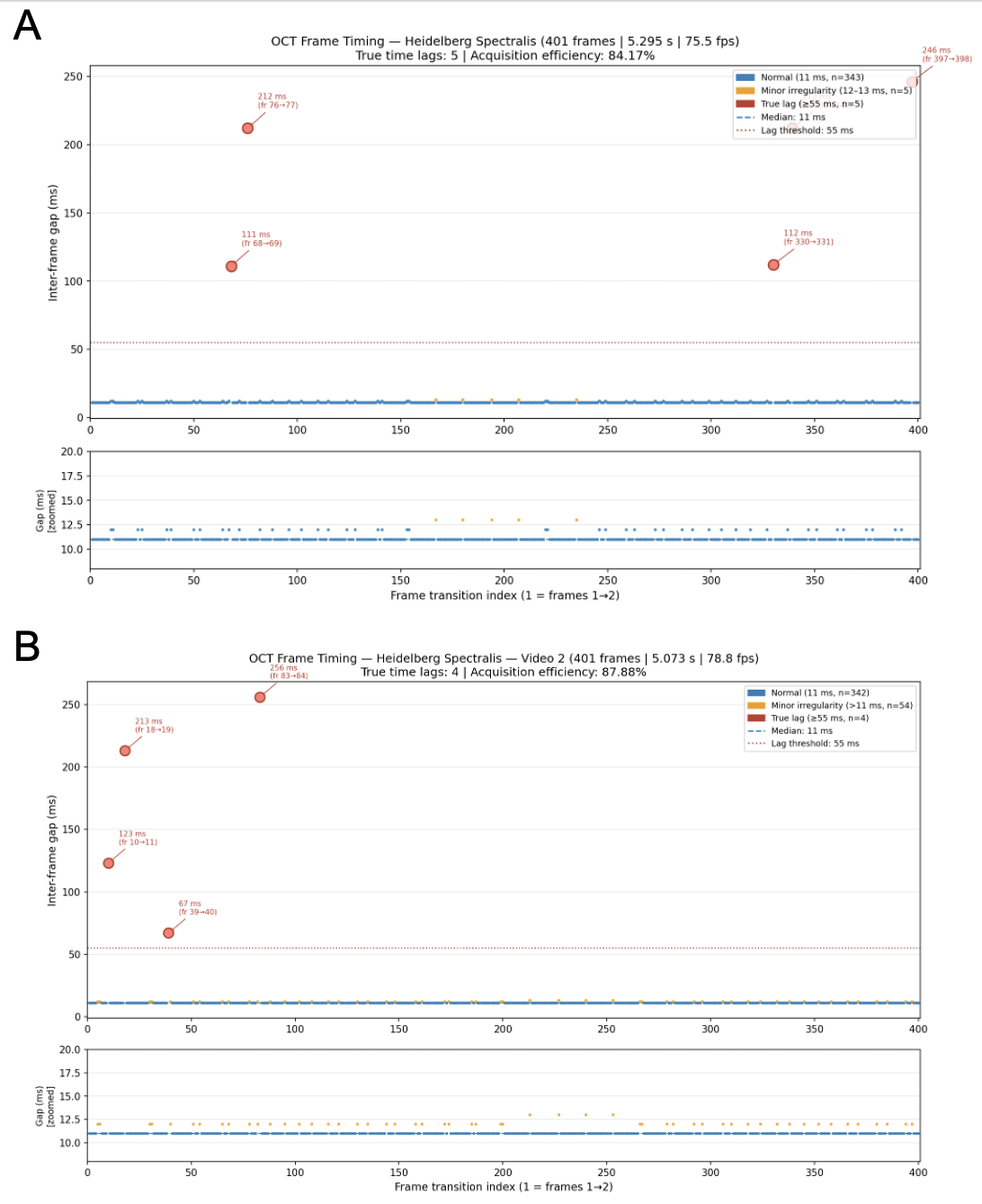


Each panel shows the full series of 400 consecutive inter-frame intervals derived from embedded DICOM timestamps (tag 0018,9074), plotted against frame transition index. The upper subplot displays the complete gap range; the lower subplot is zoomed to 8-20 ms to resolve minor irregularities. Blue dots indicate normal inter-frame intervals (11 ms, modal gap); orange dots indicate minor clock-rounding irregularities (12-13 ms), attributable to the device's millisecond timestamp precision and not physiologically meaningful; red dots indicate true temporal discontinuities (≥55 ms), consistent with transient eye-tracking correction events associated with at least one missed acquisition cycle, each annotated with its magnitude and the corresponding frame transition. The blue dashed line marks the nominal 11-ms inter-frame interval; the red dotted line marks the 55-ms discontinuity threshold (5× nominal).

In the representative arterial recording (A), five true discontinuities were identified (range: 111-246 ms), distributed aperiodically across the full 5.295-s acquisition (effective frame rate: 75.5 fps; acquisition efficiency: 84.2%). In the representative venous recording (B), four true discontinuities were identified (range: 67-256 ms), all occurring within the first 84 frames of the 5.073-s acquisition (effective frame rate: 78.9 fps; acquisition efficiency: 87.9%). Both recordings demonstrate a predominantly stable temporal cadence, with true discontinuities affecting fewer than 1.5% of inter-frame transitions.
