# Supplementary material for: Real-Time High-Resolution OCT for Imaging Retinal and Choroidal Blood Flow
Source: Invest Ophthalmol Vis Sci. 2026 May 27;67(5):69. doi: 10.1167/iovs.67.5.69 (PMC13221891; doi:10.1167/iovs.67.5.69)
Supplement: Supplement 2 [file iovs-67-5-69_s002.docx]

**Supplementary Table 1. Temporal fidelity metrics derived from per-frame DICOM timestamps in arterial and venous ART-1 movies.**

| **Category** | **Gap range** | **Artery Group** | **Vein Group** |
| --- | --- | --- | --- |
| Normal | 11 ms | 336.2 (84.1%) | 332.5 (82.6%) |
| Minor irregularity* | 12-13 ms | 48.4 (12.1%) | 50.2 (12.6%) |
| True discontinuity† | ≥55 ms | 15.4 (3.9%) | 17.3 (4.3%) |
| Time lost to true lags / movie | — | 859.4 ms (16.2%) | 646.5 ms (12.7%) |
| Effective mean frame rate | — | 74.7 fps | 78.2 fps |
| Acquisition efficiency | — | 83.8% | 87.3% |

Inter-frame intervals were classified as normal (11 ms), minor irregularity (12-13 ms), or true discontinuity (≥55 ms). Values for arterial and venous groups are reported as mean number of inter-frame transitions per movie, with percentages in parentheses. “Time lost to true lags/movie” indicates the cumulative duration attributable to true discontinuities per recording. Effective mean frame rate and acquisition efficiency are summarized separately for arterial and venous recordings.

** Minor irregularities likely reflect the millisecond precision of the timestamp encoding and were not considered physiologically meaningful.*

*† True discontinuities were interpreted as eye-tracking correction events associated with at least one missed acquisition cycle.*
